# *In situ *localization of mRNA of resembling the dirigent protein in sugarcane stems

**DOI:** 10.1186/1753-6561-8-S4-P119

**Published:** 2014-10-01

**Authors:** Paula Nobile, Juliana Mayer, Michael Brito, Izadora Pastore, Pedro Araújo, Alexandra Bottcher, Silvana Creste, Paulo Mazzafera

**Affiliations:** 1 Departamento de Biologia Vegetal, Instituto de Biologia, CP 6109, Universidade Estadual de Campinas, Campinas, SP, Brazil; 2 Departamento de Biologia, Universidade Estadual do Centro-Oeste, Guarapuava, PR, Brazil; 3 Centro de Cana - Instituto Agronômico de Campinas, C.P. 206 - CEP: 14001970 - Ribeirão Preto, SP, Brazil

## Background

The dirigent proteins (DP) families and resembling the DP (DP-like) are exclusive in land plants and related to plants defense and forming phenylpropanoids compounds optically active, mainly pinoresinol [1]. Pinoresinol is converted into a variety of lignans. The dirigent protein involvement is not confirmed in lignin formation, a biopolymer which is a negative factor for the ethanol cellulosic production. However, recently it was demonstrated *Arabidopsis *DP-like, AtDIR10, localization in the lignin polymerization site and a determinant role in the formation of a lignin specific root structure, named as *Casparian strip *[2]. Despite of the controversy of the DP forming-lignin its substrate diversity is consistent. Since DPs and DPs-*like *are represented by numerous members of gene families with high diversified sequences and with unknown functional role for most of them [3]. One of the sugarcane *DP-like*, named as *ShDP1-like*, showed special interesting due its high level of expression in the pith of mature stem [3] coinciding with higher level of the sinapyl (S) unit forming lignin [4]. Localization of DP expression to particular cell and tissue types is a necessary prerequisite in understanding the biological role of this gene.

## Methods

### In situ hybridization of mRNA

To examine the localization of *ShDP1-like *mRNA, the 191 pb PCR product obtained from the stem cDNA of IACSP04-063 sugarcane variety using the *ShDP1-like *specific pair primers was cloning at pGEM easy vector (Promega). The linearized constructed vector was used for synthesis of digoxigenin-labeled antisense and sense RNA probes with the DIG RNA Labeling Kit (SP6/T7) (Roche). 20-μm sections of paraformaldehyde-embedded sugarcane stem were subjected to *in situ *hybridization as previously described [5]. Photomicrographs were captured with an Olympus BX 51 photomicroscope equipped with an Olympus DP71.

## Results and conclusions

The *ShDP1-like *hybridization signals were detected in the vascular bundles and the fibers surround the vascular bundles in the young internode rind of the sugarcane stem, coinciding with the lignin accumulation. On the other hand, the stronger signals were detected in the parenchyma cells of mature internode pith and less pronounced signal in the vascular bundles and fibers of pith and rind cells. Interesting, unlike the parenchyma cells of young internode, the lignin accumulation appears in parenchyma cells of mature pith, and probably with a S unit-rich formation (4). Moreover, it is noteworthy that the sucrose accumulation also occurs on the parenchyma cells of mature pith. The results suggest a direct or indirect relation between *ShDP1-like *expression pattern and lignin accumulation. In order to access the *ShDP1-like *biological role, its coding sequence was isolated and cloned in overexpression vector. Currently, the transformed rice *calli *carrying the *ShDP1-like *overexpression cassette are regenerating.
